# Local bicarbonate ringer’s therapy after resection of idiopathic solitary tumoural calcinosis

**DOI:** 10.1093/jscr/rjag322

**Published:** 2026-04-28

**Authors:** Akio Sakamoto, Takashi Noguchi, Shuichi Matsuda

**Affiliations:** Department of Orthopaedic Surgery, Graduate School of Medicine, Kyoto University, Kyoto 606-8507, Japan; Department of Orthopaedic Surgery, Graduate School of Medicine, Kyoto University, Kyoto 606-8507, Japan; Department of Orthopaedic Surgery, Graduate School of Medicine, Kyoto University, Kyoto 606-8507, Japan

**Keywords:** tumoral calcinosis, solitary, bicarbonate, Ringer’s solution, surgery

## Abstract

Tumoural calcinosis is characterized by deposits of calcium phosphate crystals in soft tissue. These crystals, mainly hydroxyapatite, infiltrate tissue and make complete resection difficult because hydroxyapatite is essentially insoluble. We introduced bicarbonate Ringer’s solution (HCO₃^−^ 28 mEq/L) as local adjuvant therapy. Our patient was a 69-year-old woman with a 6-month history of a gradually enlarging solid sacral mass. After resection of the calcified material, the operative field was repeatedly washed with bicarbonate Ringer’s solution to remove residual calcium phosphate from the wall of the pseudocyst. The solution had a pH of 7.0 and an osmotic pressure of ~1.0 atm, similar to extracellular fluid. Local irrigation with bicarbonate Ringer’s solution appeared effective for removing residual deposits in idiopathic solitary tumoural calcinosis.

## Introduction

Tumoural calcinosis is characterized by solitary or multifocal deposits of calcium phosphate in extra-articular soft tissues [1]. Multifocal lesions associated with dialysis often occur around the hips, shoulders, and elbows [1]. The prevalence of tumoural calcinosis in dialysis patients has been reported to range from 0.5% to 3% [2]. Surgical resection is the primary treatment; however, recurrence may occur because complete removal can be difficult [1, 2]. We previously introduced bicarbonate Ringer’s solution as local adjuvant therapy during resection for dialysis patients with advanced tumoural calcinosis [3]. Here, we describe its use in a patient with idiopathic solitary tumoural calcinosis who was not undergoing dialysis.

## Case report

**Figure 1:**
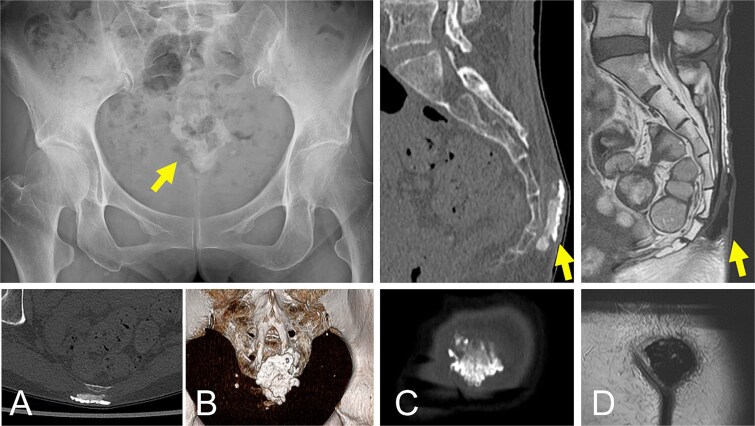
Plain radiographs (A) and CT (A-bottom, B and C) show a calcified mass in the sacral region (arrows). MRI demonstrates a lesion with low-to-high signal intensity on T1-weighted images (D) (arrows).

A 69-year-old woman presented with a 6-month history of a gradually enlarging mass in the sacral region. A slightly mobile, hard 3 × 5 cm mass was palpable. There was no redness or swelling of the overlying skin. Laboratory tests showed a leukocyte count of 5120/mm^3^ (normal range, 3500–9100/mm^3^), C-reactive protein level of 0.05 mg/dl (normal range, <0.14 mg/dl), and normal renal function (estimated glomerular filtration rate 85.2 ml/min/1.73 m^2^; normal range, <90 ml/min/1.73 m^2^). Plain radiographs and computed tomography (CT) showed an opaque, homogeneous lesion. Magnetic resonance imaging (MRI) showed low signal intensity on T1- and T2-weighted images (Fig. 1). Based on the clinical course and imaging findings, the diagnosis was solitary tumoural calcinosis.

**Figure 2:**
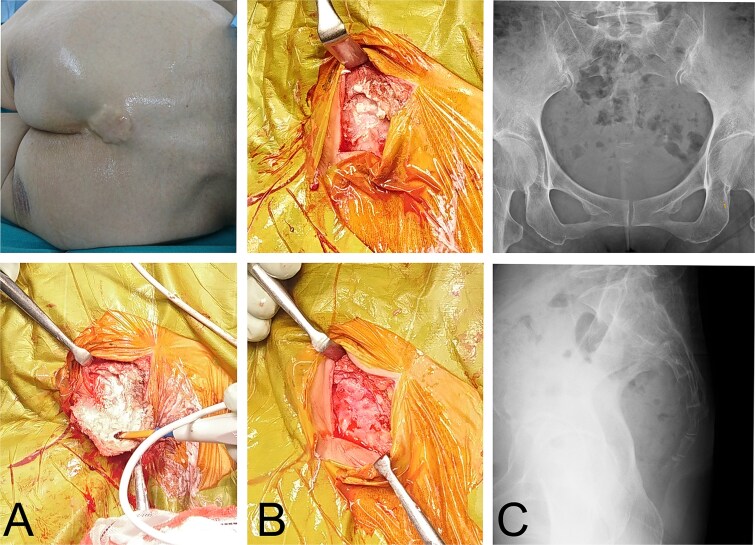
Clinical and intraoperative images. A sacral protuberance is visible (A). A white calcified lesion is exposed after skin incision (A). Residual calcified deposits on the fibrous wall are seen after resection (B) and decrease after irrigation with bicarbonate Ringer’s solution (B). Postoperative radiographs show no residual radiopaque material (C).

**Figure 3:**
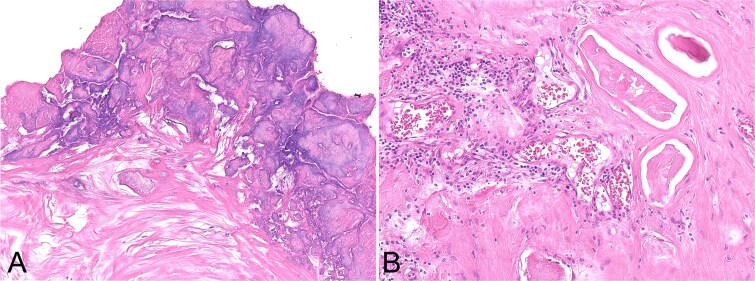
Histology shows calcified material attached to and entrapped within fibrous tissue, with an indistinct interface (A) and inflammatory cell infiltration adjacent to the calcification (B).

Under general anaesthesia, an incision was made over the lesion. The mass consisted of whitish, solid, muddy material. As much material as possible was removed; however, portions adhered to surrounding fibrous tissue and were difficult to excise. The fibrous wall was continuous with adjacent tissue without a clear border. Curettage was performed, yet whitish calcified particles remained on the fibrous wall, indicating that complete resection was not achievable. The operative field was then irrigated with 1 L of bicarbonate Ringer’s solution to facilitate removal of residual calcified material. A marked decrease in visible deposits was observed by the naked eye and fluoroscopy (Fig. 2). The postoperative course was uneventful. Histopathology showed calcified material entrapped within fibrous tissue with chronic inflammatory cells (Fig. 3). At 3-year follow-up, no mass was palpable.

## Discussion

Tumoural calcinosis typically forms nodular lesions with fibrous walls containing calcifications, macrophages, giant cells, fibroblasts, and inflammatory cells [4]. It can be primary (often associated with genetic abnormalities) or secondary, most commonly related to chronic renal failure and dialysis [1]. Repeated microtrauma has also been proposed as a contributing factor [1]. In our patient, the lesion was idiopathic and solitary; repeated mechanical stress in the sacral region may have contributed to its development.

Hydroxyapatite (Ca₁₀[PO₄]_6_[OH]₂) is a major component of tumoural calcinosis [5]. Its solubility increases in the presence of CO₂, facilitating conversion to more soluble calcium bicarbonate. Bicarbonate Ringer’s solution contains 28 mEq/L of bicarbonate ions, with a pH and osmotic pressure close to extracellular fluid, and is commonly used intravenously in clinical practice (Table 1). Based on our prior experience using bicarbonate Ringer’s solution as local adjuvant therapy in dialysis-associated tumoural calcinosis [3], we applied the same concept to idiopathic solitary tumoural calcinosis. Although solitary and dialysis-associated forms differ clinically, their calcium phosphate composition is similar; therefore, local bicarbonate irrigation may help remove residual deposits when complete resection is difficult.

**Table 1:** Bicarbonate Ringer.

Ion	mEq/L
Na^+^	130
K^+^	4
Mg^2+^	2
Ca^2+^	3
Cl^−^	109
HCO_3_^−^	28
Citrate^3−^	4

In conclusion, local irrigation with bicarbonate Ringer’s solution after resection appeared useful for reducing residual calcified material in idiopathic solitary tumoural calcinosis. Further experience is needed to clarify its effectiveness and reproducibility.
